# Rhesus monkeys learn to control a directional-key inspired brain machine interface via bio-feedback

**DOI:** 10.1371/journal.pone.0286742

**Published:** 2024-01-17

**Authors:** Chenguang Zhang, Hao Wang, Shaohua Tang, Zheng Li

**Affiliations:** 1 Center for Cognition and Neuroergonomics, State Key Laboratory of Cognitive Neuroscience and Learning, Beijing Normal University at Zhuhai, Zhuhai, People’s Republic of China; 2 IDG/McGovern Institute for Brain Research, Beijing Normal University, Beijing, People’s Republic of China; 3 Institute of Big Data and Artificial Intelligence, China Telecom Corporation Limited Beijing Research Institute, Beijing, China; 4 School of Systems Science, Beijing Normal University, Beijing, China; 5 International Academic Center of Complex Systems, Beijing Normal University, Zhuhai, China; Institute of Psychology Chinese Academy of Sciences, CHINA

## Abstract

Brain machine interfaces (BMI) connect brains directly to the outside world, bypassing natural neural systems and actuators. Neuronal-activity-to-motion transformation algorithms allow applications such as control of prosthetics or computer cursors. These algorithms lie within a spectrum between bio-mimetic control and bio-feedback control. The bio-mimetic approach relies on increasingly complex algorithms to decode neural activity by mimicking the natural neural system and actuator relationship while focusing on machine learning: the supervised fitting of decoder parameters. On the other hand, the bio-feedback approach uses simple algorithms and relies primarily on user learning, which may take some time, but can facilitate control of novel, non-biological appendages. An increasing amount of work has focused on the arguably more successful bio-mimetic approach. However, as chronic recordings have become more accessible and utilization of novel appendages such as computer cursors have become more universal, users can more easily spend time learning in a bio-feedback control paradigm. We believe a simple approach which leverages user learning and few assumptions will provide users with good control ability. To test the feasibility of this idea, we implemented a simple firing-rate-to-motion correspondence rule, assigned groups of neurons to virtual “directional keys” for control of a 2D cursor. Though not strictly required, to facilitate initial control, we selected neurons with similar preferred directions for each group. The groups of neurons were kept the same across multiple recording sessions to allow learning. Two Rhesus monkeys used this BMI to perform a center-out cursor movement task. After about a week of training, monkeys performed the task better and neuronal signal patterns changed on a group basis, indicating learning. While our experiments did not compare this bio-feedback BMI to bio-mimetic BMIs, the results demonstrate the feasibility of our control paradigm and paves the way for further research in multi-dimensional bio-feedback BMIs.

## Introduction

Brain machine interfaces (BMI), or brain computer interfaces (BCI), connect brains to machines or computers. Here we focus on a subset of such systems which use intracortical neuronal recordings from invasive electrodes implanted into cortex. Such systems can control robotic arms [1–4], cursors on computers [5–8] or tablets [9], exoskeletons [10], and paralyzed limbs via functional electrical stimulation [11–14]. Some early work related to invasive BMI recorded from single or a few neurons [1, 15, 16] and depended on learning by the user to control the activity of recorded neurons. Thanks to the development of electrode array hardware, chronic neuronal ensemble recordings have become viable [17], motivating more complex decoding methods.

These methods hold various assumptions to leverage multi-channel neural activity to offer intuitive and higher dimensional control: the population vector algorithm [18] assumes neuronal tuning can be described by preferred directions; linear filters (including the discrete Wiener filter) allow asymmetrical preferred direction distributions but assume linear tuning [19–21]; Kalman filters [22, 23] assume a state-space Markovian model with linear state transitions and linear tuning. More recent methods acknowledge the stochasticity of neuronal firing and the complexity of the cortical-musculature pathway and of the dynamics-to-kinematics relationship: particle filters [24], recurrent neural networks [25–27], support vector regression [25, 28, 29], autoregressive moving average [30], kernel autoregressive moving average [31], unscented Kalman filters [6], specialized Bayesian filters [22, 32, 33], and point process filters [34–36]. They utilize complex algorithms to mimic, in a supervised-learning approach, the natural relationship between motor cortical activity and end-effector movement, i.e. neural tuning. The approach is based on the belief that movement intentions can be accurately decoded after the decoder parameters are fitted using data recorded from actual limb movements or some reasonable substitute (when these are not available). The primary advantage of this bio-mimetic approach is intuitive and immediate control: after a brief period of model fitting and user orientation, the BMI system “plugs in”, bypassing the existing motor system.

While this kind of BMI decoder may have good initial performance, research [37–40] has found that, after practicing with neural control, users can perform better when feed-back learning is available (the work of [37] suggests focus on proprioceptive feedback).

However, the increasingly complex and often non-linear transformations used in bio-mimetic decoders may hamper, in the long-run, the ability of the user to learn these systems [41]: users’ trial-and-error strategy might work well for a simple neuronal activity to end-effector movement relationship, but not as well for complex or non-linear decoders which may include probabilistic tuning models, deep neural networks, or models that mimic the physical properties of limbs.

On the other hand, technical improvements in the stability of chronic recordings make the bio-feedback BMI paradigm increasingly feasible. A novel appendage may not need complex bio-mimetic neural tuning; humans may learn to use it from scratch, similar to how infants learn to control their limbs. To test the feasibility of this approach, we tasked monkeys to learn to control a cursor via a relatively simple control system by trial and error. Such a system may have lower initial accuracy when compared to state-of-the-artbio-mimetic BMIs, but after long-term user learning, it may offer competitive control accuracy. Compared to the bio-mimetic approach, there is much less work in this area [11, 42–45].

We are interested in simple control rules with simple assumptions, like the early work of Fetz [1] and Kennedy et al. [16]. Inspired by the four directional keys on a computer keyboard, used daily by many people to control a novel end-effector (the computer cursor), we designed and implemented an algorithm, called group weight, which sums spike counts within groups of neurons and converts the sums to cursor speed by multiplying with coefficients (weights). We use the summed firing rates of groups of neurons, since firing rates of individual neurons are variable [41]. Our algorithm does not require the sophisticated parameter estimation efforts of bio-mimetic BMI decoder training; however, analysis of neuronal tuning (preferred directions) can help us choose neuron groups so that neurons between groups are less likely to fire together, which helps facilitate separate control of two dimensions initially. In this first step in testing the feasibility of this approach, we do not compare against state-of-the-art bio-mimetic decoders here, nor attempt to solve the plethora of issues related to long-term BMI control, but rather focus on demonstrating the ability of BMI users to learn to use our bio-feedback control system. We also aim to demonstrate control of multiple dimensions simultaneously; here as an initial step, we aim for two-dimensional control.

We conducted experiments with two Rhesus monkeys to demonstrate the ability to simultaneously control two dimensions and ability to improve performance with learning. They monkeys were able to control the 2D cursor. Our analyses show task performance significantly improved over approximately one week of practice. The trajectories become straighter, consistent with learning. We analyzed group-wise neural ensemble activities across the training period and found that variability in the output-potent direction increased, supporting the presence of learning to use this algorithm on a group level. We then analyzed individual neuron’s tuning properties and found their preferred direction (PD) changed in ways that were suitable for the algorithm, indicating the neurons’ contribution to learning the new algorithm.

Even though our experiments here were not of sufficient length to allow monkeys to gain high-accuracy control, our study demonstrates the feasibility of the group weight method and suggests it warrants further investigation, both as an approach in designing BMIs and as a tool to study neural changes during learning.

## Materials and methods

### Algorithm design

#### Group weight algorithm design

The group weight algorithm converts neurons’ firing rates to the velocity of the cursor using a simple transformation. For 2D control, the conversion relies on four groups corresponding to up, down, left, and right movement, with a pair of opposing groups per dimension similar to natural flexor and extensor muscles. Firing rates of neurons in a group are summed and normalized to obtain an action value. For each dimension, we use the net action value, the positive direction action value minus the negative direction action value, to drive the cursor:

$$a_{k}=max(\frac{(\sum_{i=1}^{n_{k}}fr_{i})-\mu_{k}}{\delta_{k}}+c,0)$$ (1)

$$v_{x}=w\left(a_{1}-a_{2}\right)$$ (2)

$$v_{y}=w\left(a_{3}-a_{4}\right)$$ (3)

**Fig 1:**
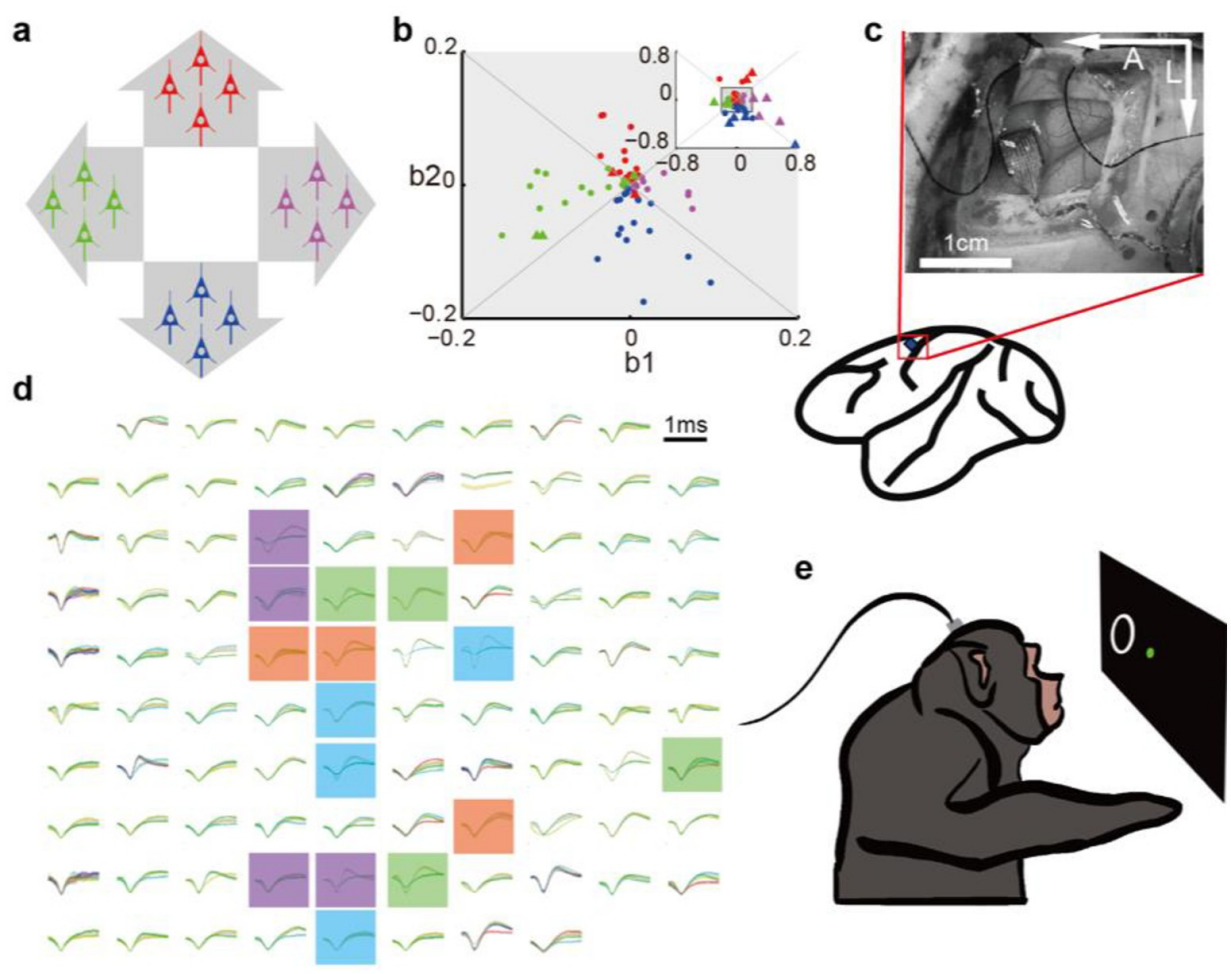
Bio-feedback BMI paradigm, array implantation, and neuronal signals. a. Schematic illustration of groups. The summed and normalized firing rate of each group of 4 neurons provides an action value. Four action values correspond to four opposing directions in two dimensions of cursor speed. b. Grouping neurons by preferred direction. We divide the 2D space of linear velocity encoding model coefficients (*b*_1_, *b*_2_ in Eq 4) into four quadrants, corresponding to each direction. We select neurons based on their preferred directions, encoding strength, and signal stability. Inset shows the same data plotted on a larger range so that all recorded neurons are visible. c. We implanted Utah microelectrode arrays into primary motor cortex hand representational area. Photo shows surgery for Monkey T. A: anterior, L: lateral. d. Sample spike waveforms 44 days after implantation for Monkey T. Waveforms of different colors indicate different units (for visualization only, we did not sort units for group weight control), and waveform thickness represents plus and minus one-half standard deviation. Panels are placed according to positions on the Utah array (wire bundle at bottom). Color shading per channel indicates group assignment. e. Experimental task. The monkey sat before a screen displaying the brain-controlled cursor (green dot) and task target (white ring) and uses brain activity to control the cursor. After moving the cursor into the target, the monkey receives a water reward.

Here, *a*_*k*_ denotes the action value, the normalized group firing rate, of group *k*; *n*_*k*_ is the neuron count in this group; *fr*_*i*_ is the firing rate of the i-th neuron (Hz); *μ*_*k*_, *δ*_*k*_ are mean and standard deviation of the group’s summed firing rate, respectively; and *c* and *w* are constant values set by the experimenter (we here use 1 and 0.375, respectively). See section Normalization method for details on how the normalization parameters *μ*_*k*_ and *δ*_*k*_ were set. In Eq 2, *v*_*x*_ is the x-axis velocity of cursor (units of screen cm/s, limited to within ±15cm/s), which is computed as the difference between the 1st and the 2nd action value (similarly for *v*_*y*_, the y-axis velocity). The four groups control two dimensions of cursor movement on the computer screen (Fig 1a). The normalization aims to avoid imbalance in action value between opposing groups and handles changes in neuronal firing rate due to recording instability. Firing rates were computed using a 5-bin moving average of the binned spike count, with each bin 100ms in duration and non-overlapping.

#### Neuron grouping

Our neuron inclusion criteria were: neurons should have long-term stable recordings and their directional encoding should be relatively strong. The stability of a neuron’s signal was judged by the number of days the neuron was recorded. If a neuron was recorded for more than 5 days, we regard it as stable. We assigned neurons to groups by their preferred direction (PD). PDs were calculated from data where in the monkey used its contralateral hand to control the cursor via a joystick. The data was collected in a center-out cursor movement task before brain control sessions. The joystick position was mapped in a one-to-one manner to the cursor location on the screen. We used a linear regression (Eq 4, the *b* variables are fitted coefficients) to calculate each neuron’s prefer direction vector (*b*_1_, *b*_2_).


$$fr=b_{0}+b_{1}v_{x}+b_{2}v_{y}$$ (4)

We plot all neurons’ PDs in Fig 1b. We separate neurons according to their preferred directions into four groups by dividing the 2D space of coefficients into four equal-angled sectors, with divisions at 1/4π, 3/4π, 5/4π, and 7/4π radians (angle from origin). For each group, we selected the most strongly tuned neurons (largest magnitude of (*b*_*1*_, *b*_*2*_) vector) that had PDs within or nearby its sector. We wanted to use a large number of neurons, so as to mitigate individual neuronal variability; however, here we had to limit the number of neurons in each group because recordings were unstable, and including all recorded neurons would mean some neurons were likely to drop out during the course of the experiments, affecting the group’s firing rate sum’s distribution. We also wanted to avoid weakly tuned neurons, which are likely to have wrong estimates for PD, and when two or more such neurons with common variation are placed in different groups, their activity only contributes to the output-null space. Thus, in the experiments here, we used 4 neurons per group, a number that is not too high so that losing a neuron was highly probable, and not too low so that losing a neuron would cause a large control ability drop. Note that, while we used PD information to set the groups, this information was not used in subsequent brain control. One may ask why bother with a bio-feedback approach that requires PD estimation, which is an important concept in bio-mimetic control. The reason we used PD-based grouping is that monkeys would not participate in the experiment if initial performance was too poor. This is a quirk of animal experiments which is not expected to occur with highly-motivated human users in a clinical environment. Using groups chosen by PDs provided sufficient initial control for the animals to continue trying, though still too poor to complete the task well. In clinical practice, groups can be chosen by other approaches which do not need training data, such as singular value decomposition of the firing rate covariance matrix [46].

#### Normalization method

The normalization constants were calculated using data from 5 to 10 minutes of pre-experiment, performed before each session. We sum each group’s firing rates during the pre-experiment and calculate each group’s summed firing rate average (*μ*) and standard deviation (*σ*). Then we use these values for group firing rate normalization (Eq 1). During the pre-experiment, monkeys performed brain control of cursor via group weight, using normalization constants from the previous session. For the first session, normalization constants were calculated from data recorded while the monkey was idle.

#### Animals, surgery, and data recording

Surgical and recording methods were similar to our previous study [47]. All surgical and experimental procedures were in compliance with the United States National Institutes of Health Guide for the Care and Use of Laboratory Animals and were approved by the Institutional Animal Care and Use Committee of Beijing Normal University. Two adult male Rhesus monkeys (Macaca mulatta), weighing 7.9kg (Monkey T) and 8.1kg (Monkey K), were used in this research. They were implanted with Utah electrode arrays (96 channels, electrode length: 1.5mm. Blackrock Microsystems, USA) in their primary motor cortex (Fig 1c) approximately 15mm lateral of midline. Surgeries were performed under sterile conditions with isoflurane (2%) anesthesia according to standard Utah array implantation procedures. We began to collect 44 days after implantation (Monkey T) (sample unit waveforms shown in Fig 1d) and data 283 days after implantation (Monkey K). Monkeys previously had some practice (3 weeks for Monkey T, approximately 2 weeks for Monkey K) with the bio-feedback BMI before the collection of data presented here. This was necessary for the monkeys to become acquainted with BMI control and for development and debugging of our control software. We recorded extracellular signals using a 128-channel Omniplex A recording system (Plexon Inc, USA). For spike detection, we used threshold-crossings with threshold set to 5 standard deviations of the voltage. We used such a high threshold to obtain recordings with high signal quality, as only the largest 1 or 2 units would be detected. We did not use spike sorting in these experiments, so as to simplify correspondence of signals across days [48]. In this text, we refer to these unsorted units as neurons. Since such multi-units have firing rates which are sums of firing rates of individual neurons, and our algorithm sums firing rates in groups, this means the groups likely contain more single-units than 4.

Animals were housed and fed in accordance with the United States National Institutes of Health (NIH) Guide for the Care and Use of Laboratory Animals. Cage sizes exceeded NIH Guide standards, and cages were equipped with water dispensers and food receptacles. Animals were solo caged, and cages were kept in a temperature, humidity, air quality, air freshness, air pressure, light, and sound-controlled room, with less than 20 animals total. Animals were fed dry primate feed three times daily and fresh fruit (or dried fruit if water restricted) once daily. Animals were given unrestricted water during days not partaking in experiments and a measured quantity of water sufficient to meet survival needs during days partaking in experiments. Animal health was observed during feeding by animal care staff and prior to experiments by experimenters. Animal weight was measured regularly or before every experiment. Health was assessed by animal appearance (especially at implant locations), presence of abnormal behavior, body weight, and appearance of waste. Cages were cleaned once daily. Enrichment was provided via television. Isoflurane (2%) anesthesia was provided during surgery and ibuprofen analgesia was provided after surgery. After the study, Utah array implants were removed from both animals under anesthesia, and they were transferred to another primate research group at the same institution.

#### Task design

The two monkeys were trained to perform center-out reaching tasks (Fig 1e), both via a hand-controlled joystick held in the hand contralateral to the implant. In the center-out task, the monkey had to move the dot cursor into the ring target to obtain a water reward. Targets appeared at screen center and then at peripheral locations, so that sequential movements toward the target resulted in center-out movements. After monkeys were aquatinted with the task, the joystick was removed. During experiments, monkeys sat in a primate chair and faced a computer screen showing the task stimuli (approximately 0.75m away). Their heads were fixed but hands were free to move. The task differed slightly between the two monkeys. For Monkey K, the center and peripheral targets appeared alternately without delay, with peripheral targets appearing at random angles (uniform distribution in 0–360°), so that movements formed a center-out-and-back series. For Monkey T, only peripheral targets were shown, and cursor position was reset to the center after each trial, so movements were only outwards. These peripheral targets appeared at only four possible places, right (0°), up (90°), left (180°), and down (270°). These changes were made to facilitate analysis of learning. Additionally, we added a short, random duration (5.5–10 seconds in the first 14 sessions, and 2.5–5 seconds thereafter) delay period or freeze time to the task sequence before the movement toward each peripheral target. Monkey T could see the peripheral target and cursor, but was unable to move the cursor during the delay period. This allowed us to analyze Monkey T’s neuronal activity both during movement and during preparation. Other minor differences are given in S1 Table of the supplementary information. Trials were limited to 10–15 seconds in duration (first three sessions used 10s and remaining sessions used 15s, both excluding freeze time). If monkeys did not reach the target during that time, the trial failed. The hold time for targets was 200 milliseconds.

#### Experiment design

Prior to the experiments reported here, Monkey T had previous experience with group weight control: this consisted of 6 days, during which different groups were used compared to the data reported here, in which Monkey T did not learn to control. This was followed by 5 days with similar groups as the data reported here, during which Monkey T did not learn to control because of distraction by the presence of the joystick. Then we removed the joystick and gave Monkey T 6 days of consecutive practice, in which it did not have obvious improvement. We report here data from 8 consecutive days of practice that happened 20 days after the previous block, during which Monkey T showed improvement in task performance. Prior to the experiments reported here, Monkey K had previous experience with the group weight control paradigm on a different 2D behavioral task (16 days), but could not learn to control during that time. This monkey also had approximately two weeks of practice with brain-controlled center-out task using group weight, but did not learn to control during that time: in the first week (4 days), the monkey used different neuron groups than in the data reported here. In the second week (6 days), groups were similar but not exactly the same as the groups reported here. After these two weeks of training, there was a 3-week-pause before the collection of data reported here, which consists of 6 consecutive days of practice in which Monkey K showed improvements in task performance. Both monkeys had two practice sessions each day, with each session about 30 minutes in duration. Monkey T’s sessions were consecutive, whereas Monkey K’s training sessions were conducted separately in the morning and afternoon. Monkey T’s total trials per session ranged from 100 to 200; Monkey K’s total trials per session ranged from 150 to 400; differences were due to different task settings (see Task design). Since failed trials generally lasted longer than successful trials, each session’s total trial count would increase as the monkey learned to control. Before each bio-feedback control session, there was a 5 to 10-minute long pre-experiment portion during which we collected data to calculate the normalization constants (see Normalization method).

### Data analysis

#### Calculation of random baseline for success rate

To verify that the success rates we observed were not due to chance, we asked what the chance rate will be if neural activity is independent from visual cue. To achieve the independency, we calculated the baseline success rates by random shuffling neural activities of 10-ms bins during cursor movement portions of sessions. Then, we fed the shuffled neural data into an offline simulation of our algorithm and center-out task (with the same set of task parameters). The task design is the same as the online version, except that we fix all trial durations to 10s. Failure to reach the target and hold within 10s was recorded as a failed trial. For Monkey K, whose cursor was not moved to the center automatically after each trial, we move the simulated cursor to the center of the last target, as if it had reached the last target. We average the success rates obtained in this manner across sessions for each monkey. Due to differences in task design between monkeys, the baseline values were different between monkeys.

## Results

### Task performance

#### Overall performance

**Fig 2:**
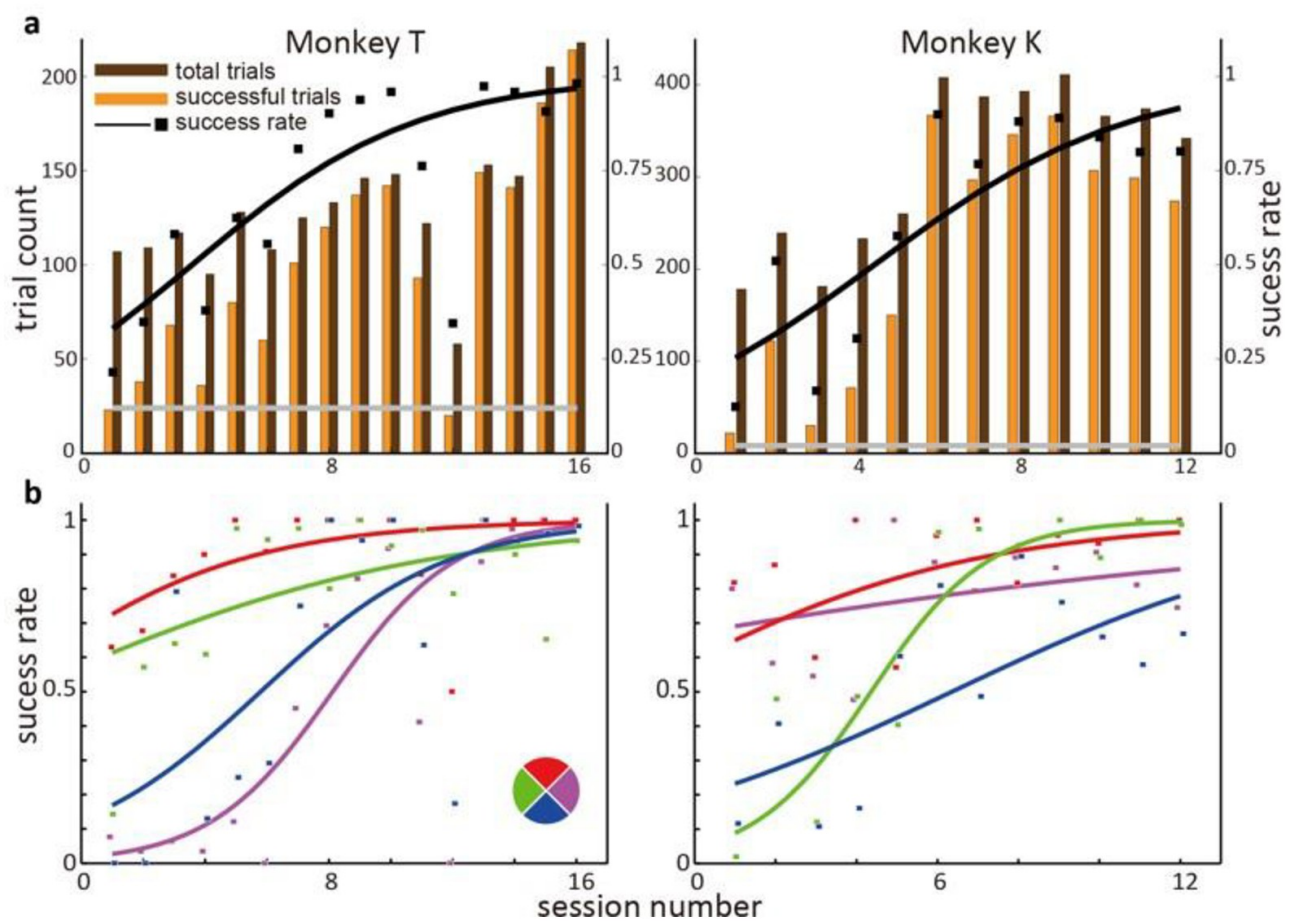
Monkey task performance. a. Trial count and trial success rate. X-axis indicates the session index (two sessions per day), and y-axis indicates the trial count (total: brown, successful: yellow) and trial success rate (black dots). Success rate data were fitted with a logistic function (black curve). Success rate was always greater than shuffled baseline (grey line). The number of successful trials and the success rate for both monkeys significantly increased with session, indicating that both monkeys could learn the group weight control paradigm. b. Success rate changes for each direction. The task of Monkey T had four possible targets; thus, we separate trials according to target. The success rate for each target increased through learning, with up and left learned early in training, whereas right and down were learned later. The task of Monkey K is not categorical, so here we divide target angles into four angular bins. Monkey K’s success rates of different directions also increase at different times in the training period.

Over approximately one week of training, monkeys’ task performance improved steadily (Fig 2a). The number of successfully finished trials increased (logistic regression *R*^2^ values MT:0.604, MK: 0.622; slope t-test p-values MT: 0.0004, MK: 0.0023) and the trial success rate increased (logistic regression R2 values MT:0.578, MK: 0.705; slope t-test p-values MT, MK<0.001). Monkey T’s success rate improved from 22% to 98%, while Monkey K improved from 12% to 80%. We obtained random baseline success rates by shuffling the neural data offline and reconstructing the control paradigm and task in MATLAB (see methods). Both monkeys’ success rates were far better than the random baseline values, which were 12% for Monkey T and 2% for Monkey K, indicating that the monkeys participated in the task and performed better than chance. Task success rate for targets in each direction significantly increased (Fig 2b). Together, these trends indicated that both monkeys successfully learned to use our group weight control paradigm.

#### Trajectories occupancy

**Fig 3:**
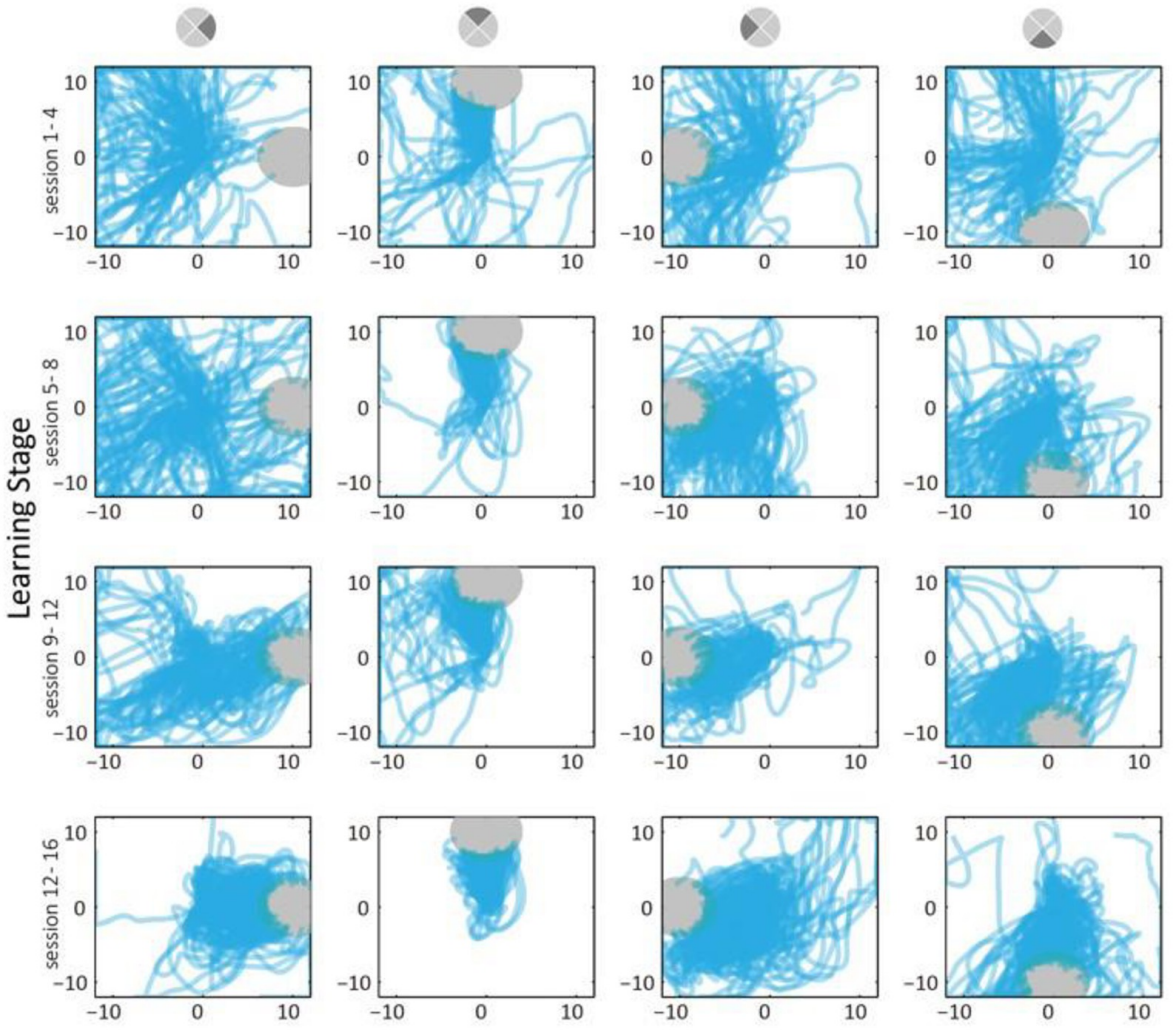
Cursor trajectories clustered through time. We plot Monkey T’s cursor trajectories from all trials (unit: cm, screen space), with sessions separated into 4 stages and trials separated by target direction. We mark targets with grey shading. In early sessions, the cursor covered similar areas for all four directions. Through learning, trajectories become more compact (e.g. up) and occupy areas not visited in early sessions (e.g. right, down). Note that up trajectories become more consistent early in the sessions, while right trajectories become better only in late sessions.

The improvement in task performance can be seen in the cursor trajectory occupancy maps (Fig 3). Here we only show Monkey T’s data, since Monkey K’s task has targets at many angles instead of just four. We segment the training into 4 stages (rows), with 4 sessions each, and separate by target direction (columns). We plot cursor trajectories (in screen space) in each panel, including both successful and failed trials. Early in the training, trajectories span almost the whole screen (Fig 3, 1st row) and occupy similar regions for different targets. The trajectories for the upward target are relatively more compact in space, indicating the monkey can move in this direction well in the early stage. As the monkey practices (row 2 and 3), the cursor trajectories each column become more compact in space. The monkey learns to move the cursor to the right half of the screen. In the late stage (row 4), movements towards each target can be seen. The trajectories show that the monkey is able to repeat existing cursor movement patterns, and create new ones: similar cursor trajectories repeat across trials (e.g. up target) and new trajectories are created (e.g. right and down). Patterns that existed at the beginning are refined earlier (e.g. up). New patterns need long term trial and error to learn and become refined later (e.g. right and down).

#### Trajectory straightness

**Fig 4:**
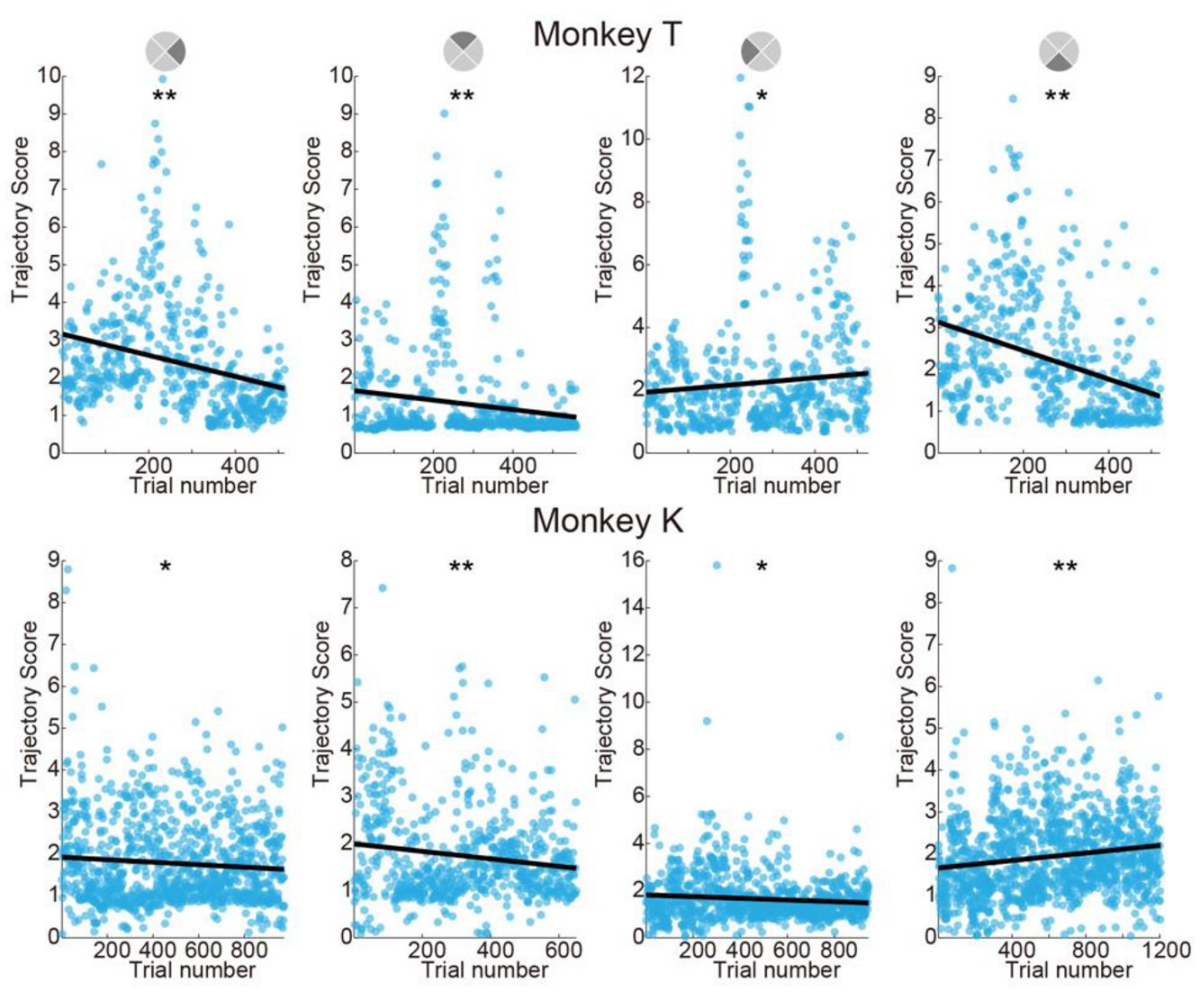
Trajectory scores decrease during learning. We separate trials according to target direction for Monkey T and according to the vector direction from cursor initial position to target center for Monkey K. The trajectory score is calculated by dividing trajectory length by the distance from cursor initial position to target center (smaller is straighter). Black lines indicate linear fits. We observed significant decreasing trends (*p < 0.05, * * *p <* 0.001), except for the left target for Monkey T and the downwards target for Monkey K.

We analyzed the straightness of cursor trajectories, a quantitative indicator of control ability. To measure straightness, we define the “trajectory score” as the length of the cursor trajectory during a successful trial divided by the length from the start point to the target center. A smaller value indicates a straighter trajectory. We separated trials according to the target direction (four quarters of the angular space, similar to those used in PD grouping: right, up, left and down). We found that both monkeys’ trajectories became straighter after learning. Monkey T’s trajectory score (Fig 4 upper) decreased in three directions (right: regression trend-line *R*^*2*^ = 0.08, slope t-test p < 10^−10^; up: *R*^*2*^ = 0.03, p < 10^−4^; down: *R*^*2*^ = 0.13, p < 10^−10^) indicating that control quality improved for these three directions. Although, the trajectories for left direction trials became slightly more curved (*R*^*2*^ = 0.01, p < 0.05, Fig 4 upper row 3rd panel), this was affected by high trajectory score trials in the late practice period and do not reflect performance decreases in this direction. For Monkey K, trajectories become straighter in three directions (right: regression trend-line *R*^*2*^ = 0.01, slope t-test p < 0.05; up: *R*^*2*^ = 0.02, p < 10^−3^; down: *R*^*2*^ = 0.31, p < 10^−7^). The task for Monkey K had targets at random angles, thus it is difficult to find clear patterns or stereotyped movements.

#### Group firing rate analysis

**Fig 5:**
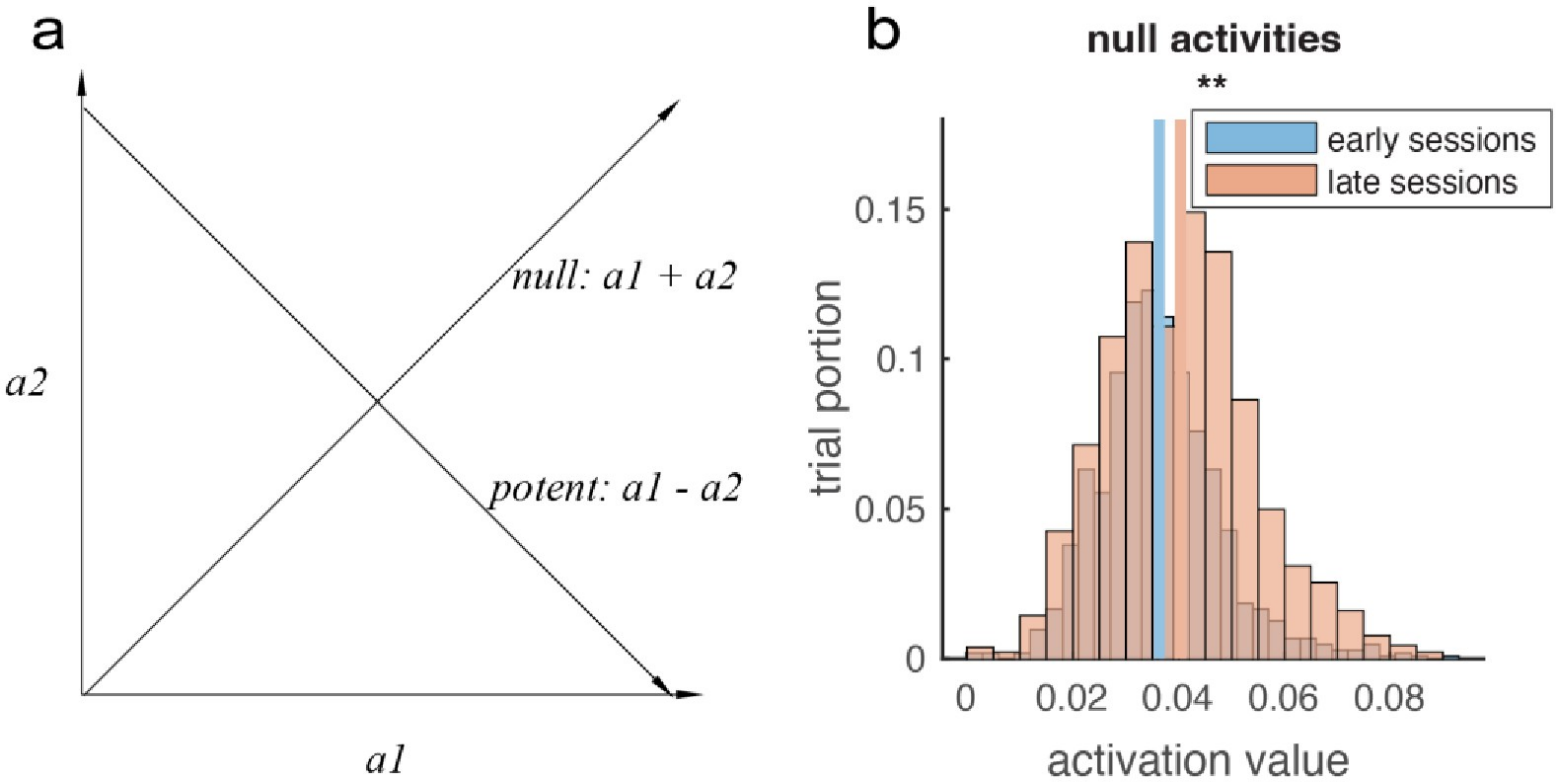
Output-null value distribution change little. a. Output-potent and output-null space illustration. In our control paradigm, the cursor speed in a dimension is proportional to the difference of two opposing action values (potent), whereas the sum of two opposing action values does not influence cursor speed (null). b. Null-space analysis of neural learning. We averaged the output-null values *a*_1_ + *a*_2_ across time in each trial, and compare the output-null values from the early sessions versus the late sessions, pooling data from two monkeys and four directions. The null component average of the late sessions was slightly larger than that in the early sessions, (ANOVA, **p < 10^−10^), this effect mostly comes from monkey T, as there was no difference in monkey K.

Our control paradigm is similar to the muscle-skeletal system in that an agonist group of neurons and an antagonist group of neurons co-activate to generate movement in a direction. When we flex our native arm, we need the agonist muscle group to activate while the antagonist group should deactivate (or have lower activation); the difference in activation of these two groups results in a net force, and thus influences the movement (output-potent), while the sum of activations does not (output-null). Thus, for our paradigm, we are interested in whether there were changes in values in the output-potent versus output-null space (Fig 5a) after learning. We use the collected neural data and offline-reconstruct activation values; We calculate the output-potent values as the group activation subtraction between the toward-target direction vs away-target direction. The output-null values are the summations of the two groups’ activation values. We pooled four directions and two monkeys’ data together and compare the output-null and output-potent values from the first sessions versus late sessions, to show the learning effect. Specifically for monkey K, we analyzed portions of successful trials in which cursor speed was medium, so as to exclude times when the monkey was not engaged in the task (idling) or when there were fast movements due to artifacts or non-task-related physical behavior. The criterion was: cursor speed between 25 to 75 percent of the maximum speed. For the output-null values, the later sessions showed larger average values than the early sessions (Fig 5b). However, this effect only existed for monkey T. For monkey K, the averages of output-null activity in the first and last sessions are the same. We believe this could be due to the slight differences in experimental paradigm.

**Fig 6:**
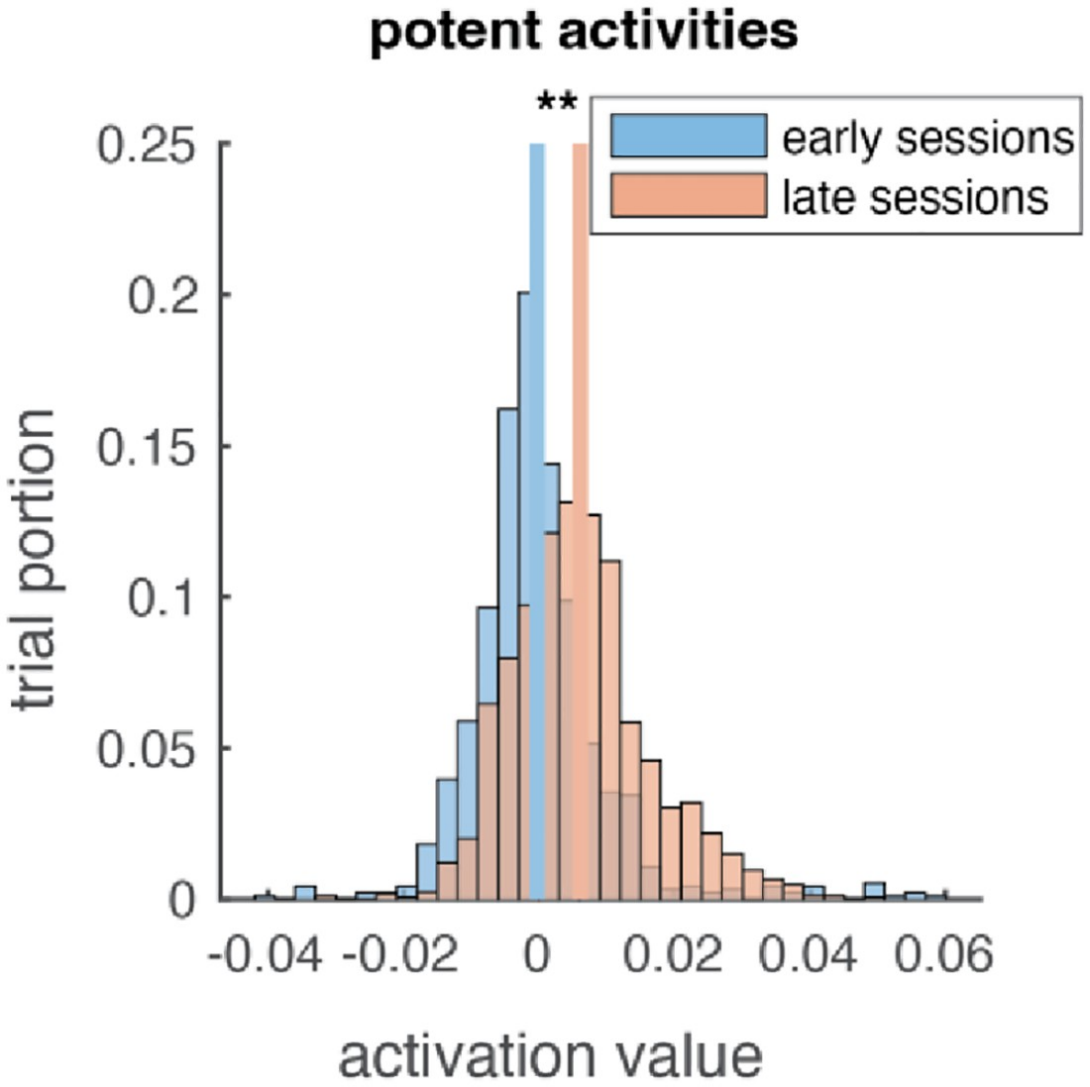
Output-potent activity increase significantly. We calculated the output-potent *a*_1_ − *a*_2_ value for each monkey and each directions and pool the data. The output-potent distribution for both monkeys changed to more positive from early (blue) to late (red) sessions. Lines indicate distribution mean (horizontal placement). (ANOVA, * * *p <* 10^−10^).

In our control paradigm, only when the output-potent values are larger than 0 could the cursor move towards the target. As seen in Fig 6, the output-potent values increased in late sessions compared with early sessions. In early sessions the output-potent value was near 0, indicating that the monkeys’ effort did not result in effective movements. Near the end of the sessions, the output-potent values are larger than 0, indicating that the group activity is more effective.

#### Tuning change through learning

The group-level neural ensemble activity shows significant output-potent increase, so next we analyze where it came from. In this section, we looked through the directional tuning properties of each neuron and found that neuron tuning changes occur in a way that is suitable for using the algorithm.

In the calculation of tuning, the neuron firing rate was regressed to the target direction (for monkey K, the calculation used target direction relative to the cursor direction). We selected epochs when cursor was moving, since for monkey T there was a period when the cursor was frozen. Specifically for monkey K, we selected epochs only when the cursor speed was neither too high nor too low, similar to the above analysis.

We examined three key values, tuning depth, fit *R*^*2*^, and preferred direction (PD). The preferred direction is the direction which has the highest firing rate, and the tuning depth and *R*^*2*^ correspond to the range of tuning function as well as the goodness of tuning fitting curve. The following analysis compares between the early learning stage and the late learning stage (first and last ¼ of sessions).

**Fig 7:**
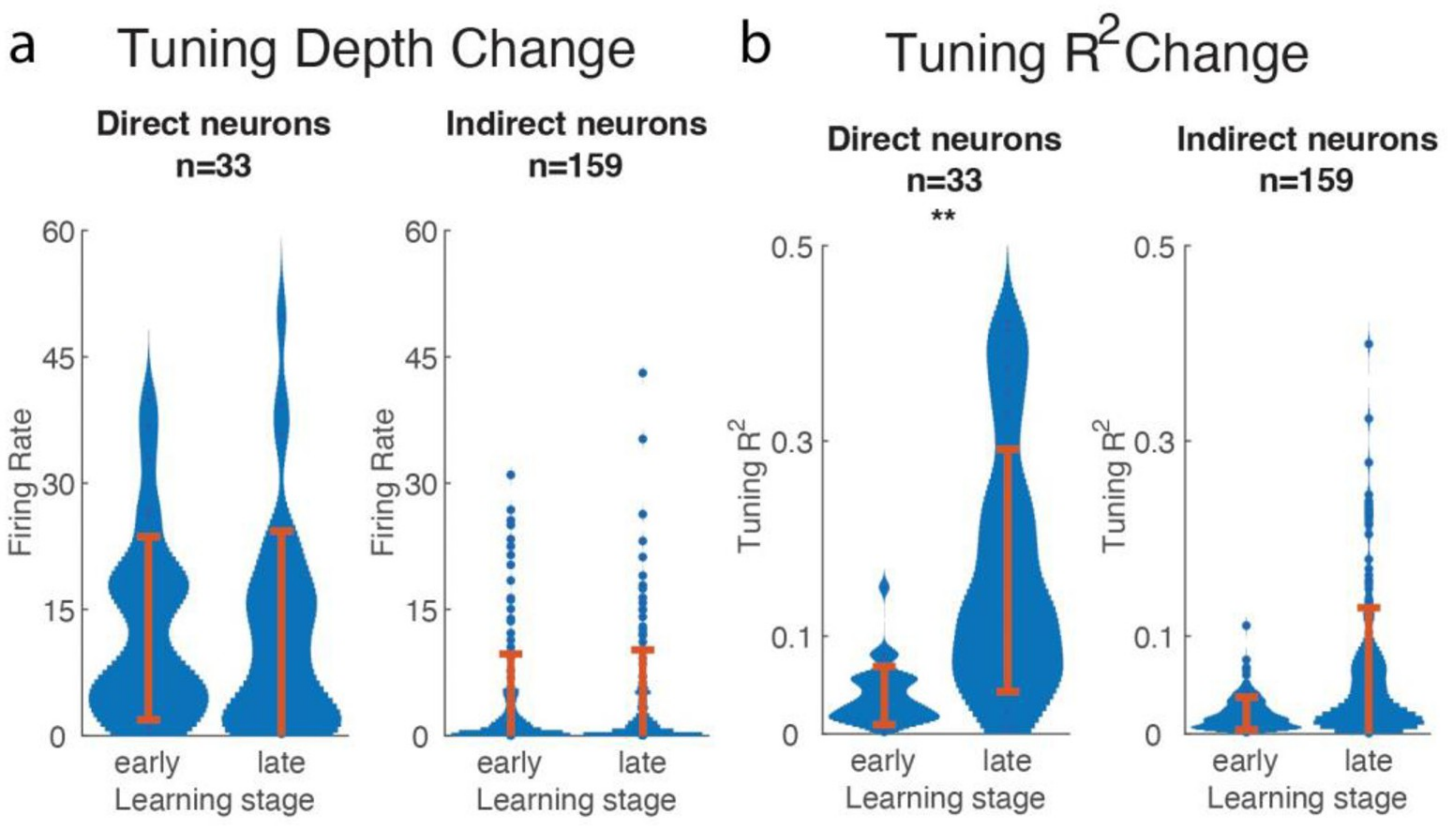
Tuning depth and *R*^2^ of direct and indirect neurons indicate learning occurred. a. Tuning depth (Hz) from early learning and late learning periods (first and last 1/4 of sessions) of direct and indirect neurons are shown. No significant difference was found (p = 0.4665, t-test). Note the difference between the direct and indirect groups is due to our neuron inclusion criteria. b. Comparison between early and late learning period shows an increase in tuning *R*^2^. The direct neurons *R*^2^ ranges from 1 * 10^−4^ to 0.63 (data pooled between monkeys).

The tuning depth did not show a significant change (Fig 7a). We separated neurons into direct neurons, whose firing rate was feed into the task, and indirect neurons, whose firing rate was not. Neither of the two groups’ tuning depths experienced a significant change. Note that the difference of tuning depth between the direct and indirect neurons is due to our neuron inclusion criteria, as we chose neurons with substantial tuning to avoid neural activity correlation in weakly-tuned neurons.

The quality of tuning, *R*^*2*^, changed during the learning period (Fig 7b), in general increasing during learning. When we compared the change within direct neurons and indirect neurons; we found that the *R*^*2*^ increased significantly (*p* = 7.93 * 10^−8^, paired-t-test) for the direct neurons, and significant (*p* = 3.43 * 10^−13^) but small changes for the indirect neurons.

**Fig 8:**
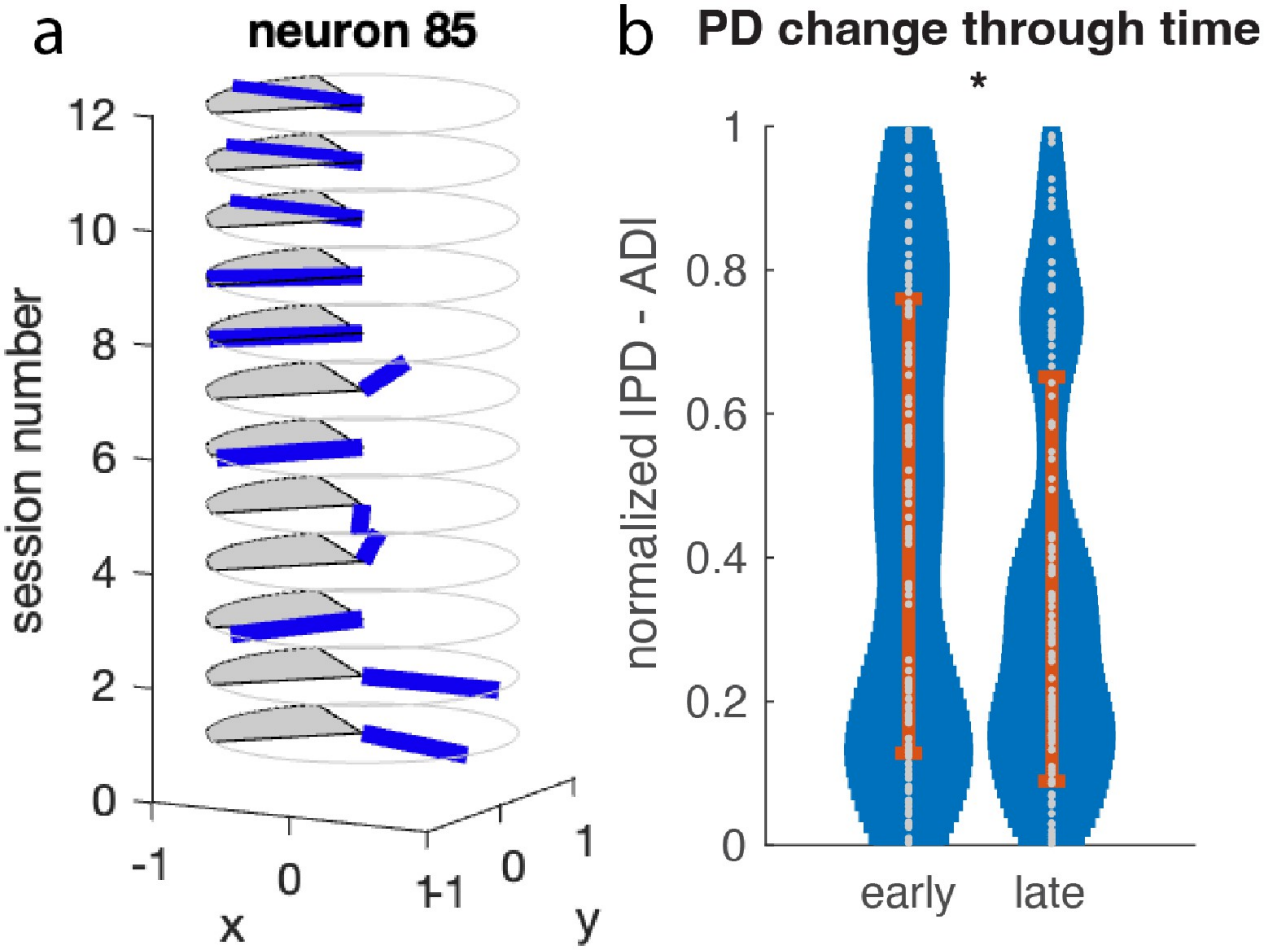
The tuning change of individual neurons. a. An example neuron with changing preferred direction. x- and y- axes are the 2D space of movements and the z-axis is session. Each disk represents a session. The blue bar on the disk represents the neuron’s preferred direction, and the shaded area on the disk represents the neuron’s assigned direction. It can be seen that the preferred direction changes towards the assigned direction. b. Changes in direct neurons’ PD over time. The PD of direct neurons in early and late learning periods are compared. Data from two monkeys and all four directions are pooled together. We calculate the normalized |*PD* − *AD*|, which represents the directional difference between the neurons’ PD and their assigned direction (AD). It is 1 for opposite directions and 0 for the same direction. When comparing early and late learning stages, this value becomes smaller, indicating that the tuning is shifting towards the assigned direction.

In our group weight method, we group neurons into four groups, assigning each group to one direction as their contribution direction. We wanted to know whether learning changed the neurons’ PD towards their assigned direction (AD). In Fig 8a, an example neuron is shown. For this specific neuron, the PD changed towards the AD.

Next we calculated the PD for all direct neurons and compared to their AD. The analysis showed that neurons’ tuning PD changed towards the AD (Fig 8b). Here we compared the early and late sessions for both monkeys, and plot normalized direction difference between PD and AD, where 1 means they are opposite, and 0 means they are the same. For neurons from both monkeys, the normalized direction difference decreased significantly (z-test, n = 33, *p* = 0.012), which is consistent with our expectation (for more examples of PD change, see S1–S3 Figs).

## Discussion

In this study, monkeys used a bio-feedback BMI based on a relatively simple control law that converts neuronal activity to 2D cursor movement. Over about a week’s practice, two monkeys improved their BMI control ability, increasing trial success rate by around 70%. We presented evidence in terms of both behavioral and neuronal activity which supports the occurrence of learning.

Related to our work are studies such as those by Kennedy et al. [16]. Their work relied on a single neuron to control each dimension, with firing rate change mapped to single-directional velocity, combined with position reset (to edge of screen) on click. Arduin et al. [44] proposed a bi-directional control method for one dimension and showed that the controlling neuron changed in firing rate differently from nearby, non-controlling neurons. Law et al. [45] showed that control accuracy of a bio-feedback decoder increases with the number of neurons. Like them, we use the combined firing rates of neurons in a group. Another related study was done by Moritz et al. [11], who used a relatively simple control method to build a functional electrical stimulation based neural bypass. Our work uses a somewhat similar neural-movement mapping as the above studies, but we control 2 dimensions using opposing groups of neurons. The opposing group mechanism lets us analyzed changes in the output-potent and output-null directions (Figs 5 and 6). The 2-dimensional control allows us to examine trajectories in 2D space (Fig 3) and analyze path straightness (Fig 4).

Readers might be interested in the difference between our control method and the population-vector algorithm [18]. Our method assigns neurons to groups that have orthogonal contribution directions, thus each neuron only influence one direction. We also do not normalize each neuron’s firing rate individually, but rather collectively in a group-wised manner. The reason for this is to decrease neurons’ firing rate variability by leveraging a group of neurons. We also use a threshold to remove the effect of small firing rates. Our method will have a same effect as the population vector algorithm under the case when the neurons’ preferred directions are evenly distributed among the four cardinal directions and have the same tuning depth. Our main goal when designing this group weight method is to minimize the assumptions on neural activity, to build a simple algorithm and let the bio-feedback learning take charge. The settings of groups and thresholds were done to avoid unintentional movement caused by neural firing variability.

### BMI learning

The initial performance after the monkeys started using brain control is low, although higher than random control (Fig 2), indicating the monkeys already had some level of control. However, the performance did not approach saturation (in terms of trial success rate) until late in training after several sessions. Some studies in the field focus on the progression of neural learning of BMI mappings [49–51]. They perturb the well-learned BMI mapping by re-assigning the decoding parameters and found that monkeys’ performance increased within one session [49, 50] or across sessions [51, 52].

In comparison, our work instead focuses on developing one simpler mapping between neural activity and movements, thus we did not train monkeys to familiarize with other BMI mappings beforehand, but only with the one group weight mapping. Thus, unlike their study, we did not compare neural activity by projecting on the original mapping (there was none); however, the cursor trajectories show that the monkeys achieve control of the new mapping (Fig 3).

We calculated neurons’ PD by assuming cosine tuning. After fitting neural activity and the intended movement direction using a cosine, we obtained the peak direction (PD) the range of firing (tuning depth), and the quality of the fitting (R2). In each of the monkeys’ training sessions, PDs are ever-changing during learning, which is in line with previous work [38]. We also found that the tuning R2 increased, which is consistent with their results.

Besides measuring tuning properties, we also analyzed group level neural activity in terms of output potent-and-null-space (Figs 5 and 6) and found changes over time. The sum of activity of agonist and antagonist groups (output-null value) increased during learning in one monkey but not the other. This could be an effect of the task design. The difference of activity of the agonist and antagonist groups (output-potent value) consistently increased in both monkeys. This is consistent with the fact that both monkey’s control performance improved; the performance increase is explained by changes in the group-level activity in terms of output-potent space.

### Bio-mimetic and bio-feedback BMI

While bio-mimetic BMIs focus on the decoder mimicking the neural activity and movement relationship, bio-feedback BMIs focus on the user’s feedback learning. Methods actually fall into a spectrum between bio-mimetic and bio-feedback, with some work relying on aspects of both, for example, using a linear decoder as in a bio-mimetic way, but also relying on bio-feedback to let the user increase performance. Ganguly and Carmena [39] showed that monkeys could learn to control a cursor using different sets of randomly-chosen linear filter parameters and switch readily between them, showing the strong adaptive capacity of the motor cortex. Balasubramanian et al. [4] showed that over long-term training, functional neuronal connections changed, suggesting long-term training may alter the motor cortex. Here, our method relies on a minimum of parameters, by assigning neurons to groups (though for monkey experiments, this assignment was made intelligently to promote engagement) instead of finding the parameters for each neuron, relying instead on bio-feedback for performance increase. Our study has limitations. One was instability in neural recording, which limited our ability to extend the training period. Our experiments were perhaps too short to see substantial changes in neuronal activity patterns. Since we did not spike sort, recording instability may have meant some neurons were included in a group in only a subset of sessions, which meant the group firing rate statistics changed between sessions. We tried to compensate for this using the normalization procedure, but the effect of recording instability is still visible (e.g. session 12 in Fig 2a left panel). Another area for improvement is the selection and grouping of neurons. We grouped based on preferred direction, which may be less optimal than using a method that considers existing connectivity structure, which can determine what is easily learnable [53, 54], at least in the short-term. While the ultimate goal of building bio-feedback BMIs with minimum parameters is to allow high control accuracy, we did not train monkeys long enough for learning to increase performance to a level comparable with bio-mimetic methods. This study was meant as a feasibility demonstration, a first step showing that simple, group-based method, for multi-dimensional control is learnable. Tweaking of the control law for better performance and longer duration training are needed in future studies.

## Conclusion

We implemented the group weight method as a bio-feedback BMI control paradigm with simple assumptions. Through one week of training, monkeys showed increases in success rate and trajectories become straighter, indicating learning occurred. Group-based and single neuron metrics also indicated learning occurred. This simple bio-feedback control paradigm has potential to control multi-dimensional cursors or robotic limbs.

## Supporting information

S1 Fig Neuronal PD change across learning, monkey T. Each column represents a group of neurons and each pie plot corresponds to a session. In each pie plot, the shaded area shows neurons’ assigned direction (AD) and the colored bars show neuron’s tuning (PD). In early learning (session 1), the neurons’ PDs are not close to their AD (except for group 3). However, in the late sessions (15 and 16), neurons’ PDs are closer to the AD.
(DOCX)

S2 Fig Neuronal PD change across learning, monkey K. Same notation as S1 Fig. (DOCX)

S3 Fig Distance between neuronal PD and assigned directions AD across learning, both monkeys. X-axis is time (session number) and Y-axis is the normalized |PD—AD|, which is 1 for opposite direction and 0 for same direction. Thus, the directional difference is mapped from [-π, π] to [0,1]. The sector of the assigned direction [-45°, 45°] (shaded sector in S1 Fig) is mapped to [0,0.25], and values lower than 0.5 indicate the channel contributes to the direction of movement. Most channels’ |PD–AD| is smaller than 0.5, showing that they contribute to the movement direction.
(DOCX)

S1 Table Task difference between the two monkeys. (DOCX)
