# Supplementary material for: Rhesus monkeys learn to control a directional-key inspired brain machine interface via bio-feedback
Source: PLoS One. 2024 Jan 17;19(1):e0286742. doi: 10.1371/journal.pone.0286742 (PMC10793883; doi:10.1371/journal.pone.0286742)
Supplement: S1 Fig — Each column represents a group of neurons and each pie plot corresponds to a session. In each pie plot, the shaded area shows neurons’ assigned direction (AD) and the colored bars show neuron’s tuning (PD). In early learning (session 1), the neurons’ PDs are not close to their AD (except for group 3). However, in the late sessions (15 and 16), neurons’ PDs are closer to the AD. (DOCX) [file pone.0286742.s001.docx]

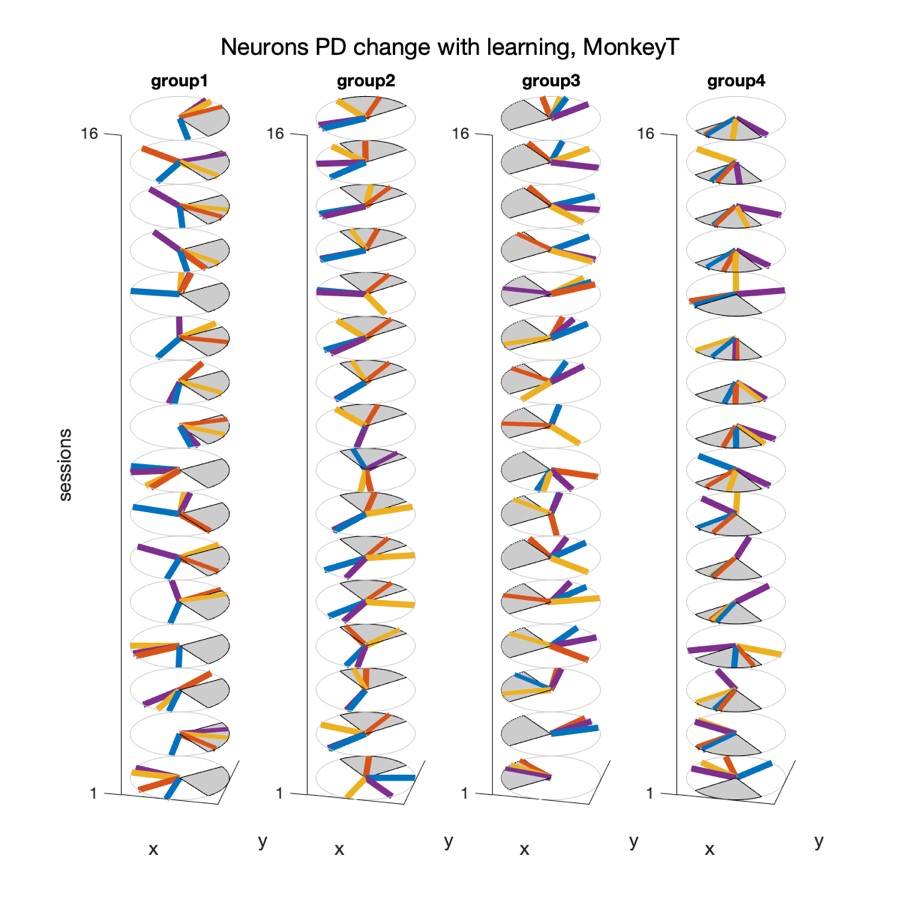


**Fig S1. Neuronal PD change across learning, monkey T.** Each column represents a group of neurons and each pie plot corresponds to a session. In each pie plot, the shaded area shows neurons’ assigned direction (AD) and the colored bars show neuron’s tuning (PD). In early learning (session 1), the neurons’ PDs are not close to their AD (except for group 3). However, in the late sessions (15 and 16), neurons’ PDs are closer to the AD.
