# Supplementary figures and images for: Rhesus monkeys learn to control a directional-key inspired brain machine interface via bio-feedback
Source: PLoS One. 2024 Jan 17;19(1):e0286742. doi: 10.1371/journal.pone.0286742 (PMC10793883; doi:10.1371/journal.pone.0286742)

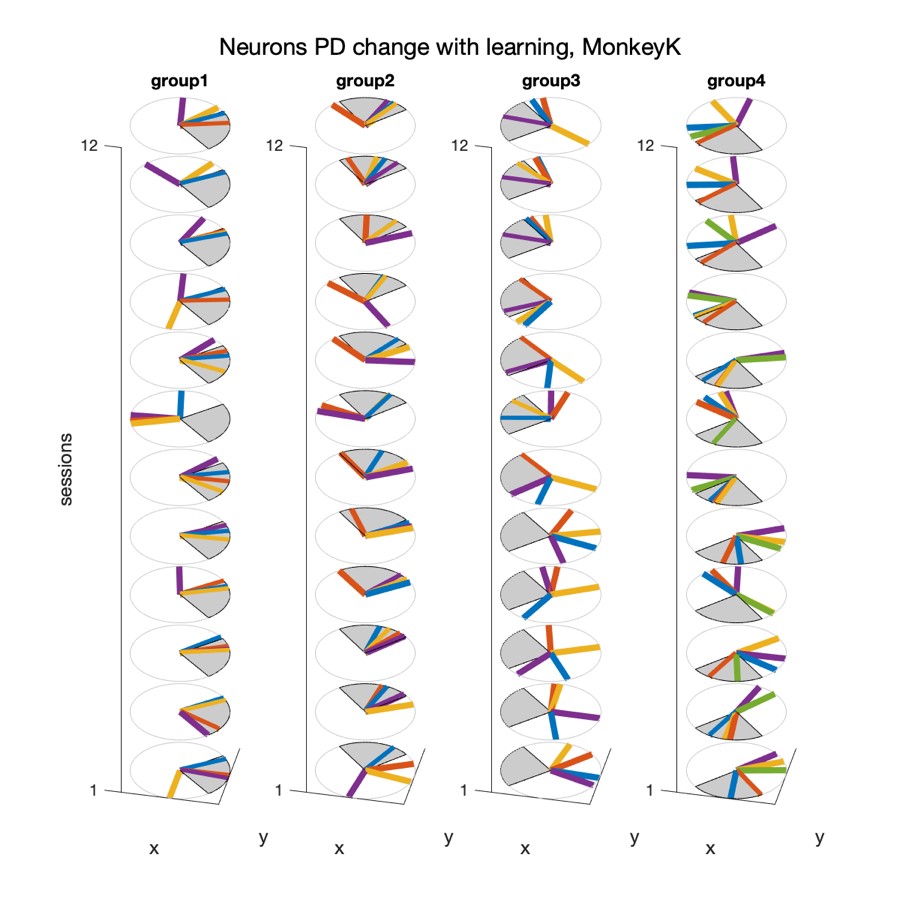


**Fig S2. Neuronal PD change across learning, monkey K.** Same notation as Fig S1.

Supplement: S2 Fig — (DOCX) [file pone.0286742.s002.docx]
