# Supplementary material for: Rhesus monkeys learn to control a directional-key inspired brain machine interface via bio-feedback
Source: PLoS One. 2024 Jan 17;19(1):e0286742. doi: 10.1371/journal.pone.0286742 (PMC10793883; doi:10.1371/journal.pone.0286742)
Supplement: S3 Fig — X-axis is time (session number) and Y-axis is the normalized |PD—AD|, which is 1 for opposite direction and 0 for same direction. Thus, the directional difference is mapped from [-π, π] to [0,1]. The sector of the assigned direction [-45°, 45°] (shaded sector in S1 Fig) is mapped to [0,0.25], and values lower than 0.5 indicate the channel contributes to the direction of movement. Most channels’ |PD–AD| is smaller than 0.5, showing that they contribute to the movement direction. (DOCX) [file pone.0286742.s003.docx]

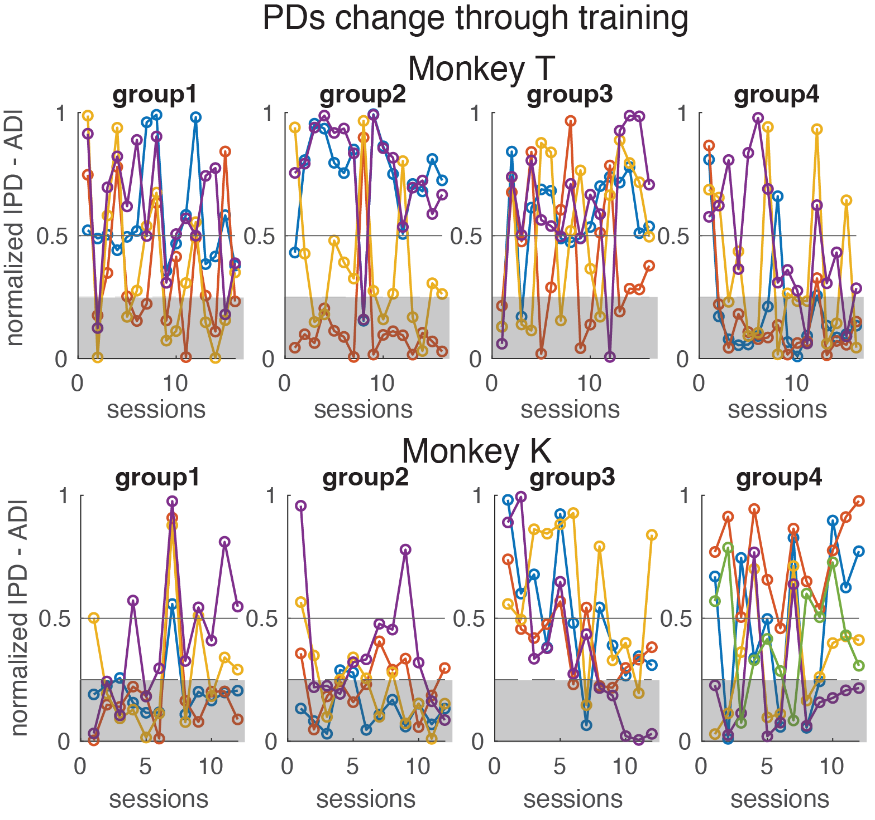


**Fig S3. Distance between neuronal PD and Assigned directions AD across learning, both monkeys.** X-axis is time (session number) and Y-axis is the normalized |PD - AD|, which is 1 for opposite direction and 0 for same direction. Thus, the directional difference is mapped from [-π, π] to [0,1]. The sector of the assigned direction [-45°, 45°] (shaded sector in Fig S1) is mapped to [0,0.25], and values lower than 0.5 indicate the channel contributes to the direction of movement. Most channels’ |PD – AD| is smaller than 0.5, showing that they contribute to the movement direction.
