# Supplementary material for: Rhesus monkeys learn to control a directional-key inspired brain machine interface via bio-feedback
Source: PLoS One. 2024 Jan 17;19(1):e0286742. doi: 10.1371/journal.pone.0286742 (PMC10793883; doi:10.1371/journal.pone.0286742)
Supplement: S1 Table — (DOCX) [file pone.0286742.s004.docx]

**Table S1. Task difference between the two monkeys.**

|  | **Monkey K** | **Monkey T** |
| --- | --- | --- |
| Freeze duration | N/A | 5.5-10s or 2.5-5s |
| Targets | Center and peripheral | All peripheral |
| Target angle | [0, 360) | 0,90,180,270 |
| Peripheral targets distance | 15cm | 10cm |
| Target diameter | 10cm | 8cm |
| Cursor speed coefficient (w) | 15 cm/s | 10 cm/s |
| Cursor range | [-15cm, 15cm] | [-12cm, 12cm] |
